# Assessing cardiovascular risk in chronic kidney disease patients prior to kidney transplantation: clinical usefulness of a standardised cardiovascular assessment protocol

**DOI:** 10.1186/s12882-017-0795-z

**Published:** 2018-01-08

**Authors:** Robin Ramphul, Maria Fernandez, Sam Firoozi, Juan C. Kaski, Rajan Sharma, Debasish Banerjee

**Affiliations:** 1 Renal and Transplantation Unit, St George’s University Hospitals NHS Foundation Trust, Blackshaw Road, Tooting, London, SW17 0QT UK; 2 Cardiology Clinical Academic Group, Molecular and Cell Sciences Research Institute, St George’s, University of London, Cranmer Terrace, London, SW17 0RE UK

**Keywords:** Kidney transplantation, Cardiovascular disease, Dobutamine stress echocardiography, Coronary angiography, Cardiovascular events, Chronic kidney disease

## Abstract

**Background:**

Despite pre-kidney-transplant cardiovascular (CV) assessment being routine care to minimise perioperative risk, the utility of such assessment is not well established. The study reviewed the evaluation and outcome of a standardised CV assessment protocol.

**Methods:**

Data were analysed for 231 patients (age 53.4 ± 12.9 years, diabetes 34.6%) referred for kidney transplantation between 1/2/2012-31/12/2014. One hundred forty-three patients were high-risk (age > 60 years, diabetes, CV disease, heart failure, peripheral vascular disease) and offered dobutamine stress echocardiography (DSE); 88 patients were low-risk and offered ECG and echocardiography with/without exercise treadmill test.

**Results:**

At the end of follow-up (579 ± 289 days), 35 patients underwent kidney transplantation and 50 were active on the waitlist. There were 24 events (CV or death), none were perioperative. One hundred fifteen patients had DSE with proportionally more events in DSE-positive compared to DSE-negative patients (6/34 vs. 7/81, *p* = 0.164). In 42 patients who underwent coronary angiography due to a positive DSE or ischaemic heart disease symptoms, 13 (31%) had events, 6 were suspended, 11 removed from waitlist, 3 wait-listed, 1 transplanted and 17 still undergoing assessment. Patients with significant coronary artery disease requiring intervention had poorer event-free survival compared to those without intervention (56% vs. 83% at 2 years, *p* = 0.044). However, the association became non-significant after correction for CV risk factors (HR = 3.17, 95% CI 0.51–19.59, *p* = 0.215).

**Conclusions:**

The stratified CV risk assessment protocol using DSE in all high-risk patients was effective in identifying patients with coronary artery disease. The coronary angiograms identified the event-prone patients effectively but coronary interventions were not associated with improved survival.

## Background

Atherosclerotic coronary artery disease is a well-documented complication of renal disease with the incidence and severity increasing as the glomerular filtration rate decreases [1, 2]. Coronary angiography (CA) in asymptomatic patients with end stage renal disease (ESRD) have shown coronary artery disease (defined as luminal occlusion >50%) in between 37 and 58% [3–5]. In the general population without renal failure, it is widely accepted that percutaneous coronary artery intervention in asymptomatic and stable coronary artery disease (CAD) does not reduce mortality and may only confer a modest improvement in quality of life that dissipates over time [6–8]. These studies have largely excluded patients with severe renal failure and therefore these findings cannot be applied to these patients.

The gold standard treatment for patients with ESRD is a kidney transplantation which offers better survival and quality of life compared to other forms of renal replacement therapy [9]. However there is a significant risk of cardiovascular (CV) events during transplantation and the risk continues to be high before and after kidney transplantation [10–14]. Death has been reported as the leading cause of graft loss in patients aged above 40 years with cardiovascular disease and infection responsible for the majority [15].

Most transplant centres therefore implement a screening programme to identify asymptomatic patients with coronary artery disease and treat with pharmacological therapy, percutaneous coronary artery intervention (PCI) or coronary artery bypass grafts (CABG) to reduce CV events and exclude patients with very high risk from being listed for transplantation. The ideal approach to cardiovascular screening is unknown and differs from centre to centre. Some units have adopted a risk-stratified approach using non-invasive techniques followed by coronary angiography for high risk patients only, others offer coronary angiography to all potential transplant recipients undergoing evaluation [16–20]. However, the benefit of cardiovascular screening is unclear, particularly if this does not result in coronary revascularisation and/or leads to delays in transplantation.

The practice at our renal transplant centre is to evaluate the cardiovascular risk pre-transplantation for all potential kidney transplant recipients and offer coronary angiography only to those with symptoms of myocardial ischaemia or suggestion of cardiac ischaemia on dobutamine stress echocardiography (DSE). The purpose of this study was to evaluate the results of a standardised protocol, using DSE and CA, to screen prospective renal transplant recipients for coronary artery disease. Although we also examined the incidence of cardiac events among those who were screened, this study could not address whether screening and pre-emptive intervention reduced the rates of those events.

## Methods

**Fig. 1:**
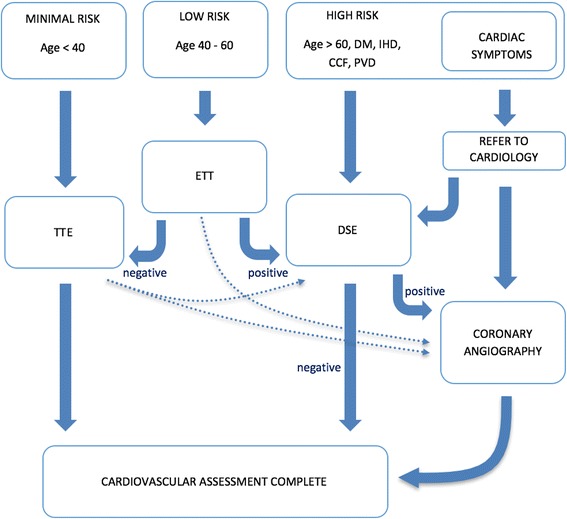
Cardiovascular risk stratification protocol. DM, Diabetes Mellitus; IHD, Ischaemic Heart Disease; CCF, Congestive Cardiac Failure; PVD, Peripheral Vascular Disease; TTE, Transthoracic Echocardiography; ETT, Exercise Treadmill Test; DSE, Dobutamine Stress Echocardiography

This study was a retrospective cohort study. Data was obtained using medical chart review. All prospective renal transplant recipients were assessed according to the same work-up protocol (Fig. 1). The clinical evaluation protocol used during the period of study was based on published recommendations from European Renal Best Practice, UK Renal Association and British Transplant Society, European Association of Urology and American Society of Transplantation [21–24]. All patients referred for evaluation for suitability for cadaveric or live-donor kidney transplantation between 1st February 2012 and 31st Dec 2014 were included. Each patient underwent cardiac risk stratification and was assigned to a ‘high-risk’ group, i.e. those older than 60 years of age or 60 and below with at least one of the following cardiac risk factors: diabetes, ischaemic heart disease, peripheral vascular disease, congestive cardiac failure; a ‘low-risk’ group i.e. those patients aged between 40 to 60 years old with none of the mentioned cardiac risk factors; and a ‘minimal risk’ group i.e. those younger than 40 with none of these risk factors. The ‘low-risk’ group adopted in the data analysis comprised patients belonging to the minimal-risk and low-risk cohorts of the protocol. Other CV risk factors such as smoking history, family history of CV disease or dialysis duration were not included in the protocol. After the risk stratification, cardiac investigations were requested according to our recipient evaluation protocol (Fig. 1); high-risk patients requiring DSE; minimal-risk patients below age 40 years undergoing transthoracic echocardiogram (TTE); and low-risk patients between ages 40-60 years requiring an exercise treadmill test (ETT). The low-risk patients with positive or inconclusive exercise treadmill test or abnormal TTE were assessed with DSE. The patients with positive DSE, symptomatic angina or acute coronary syndrome (ACS) underwent coronary angiography. If the coronary lesions were felt to be amenable to coronary stenting by the treating cardiologist, these were deployed at the time of coronary angiography. For more complex lesions, revascularisation strategy (i.e. coronary stenting, CABG or pharmaceutical therapy) was determined at a multidisciplinary meeting between cardiology and cardiothoracic specialties. The case-records of each patient were reviewed from the date of referral to the end of the study.

A positive DSE was defined as one with ≥ 2 ischaemic segments and significant CAD as coronary artery luminal stenosis ≥ 50%. Events included the standard definition for major adverse cardiovascular events and were defined as the occurrence after referral for transplant evaluation of a non-fatal cardiovascular event (myocardial infarction, unstable angina, congestive heart failure, stroke, transient ischaemic attack and amputation) and sudden cardiac death or death from any cause. Events were captured by review of patient medical records and out-of-hospital events reported by the patient to the dedicated transplant coordinator who contacts the prospective transplant recipients yearly.

The pre-transplant CV assessment is only one aspect of a holistic approach to ensuring transplant candidate suitability. Hence, successful completion of the CV assessment did not result in activation on the transplant wait-list unless all aspects of the transplant candidate work-up had been completed.

The results were analysed using the statistical software package IBM SPSS (version 20). Continuous variables were expressed as mean ± standard deviation except where stated. Annual event rate (AER) represents the proportion of patients having events per year. The difference between groups were analysed using chi-squared tests, event-free survival estimated by Kaplan-Meier method and the effect of DSE and coronary angiographic findings were assessed using the cox-proportional hazard model with correction for age, diabetes, ischaemic heart disease, hypertension, stroke or Transient Ischaemic Attack (TIA), cholesterol, Renal Replacement Therapy (RRT) modality and cardiac medication. Results with a *p* value less than 0.05 were considered significant. The study was approved as an audit by the St George’s Clinical Audit department and hence exempted from formal ethics approval process.

## Results

### Characteristics of patients screened

**Fig. 2:**
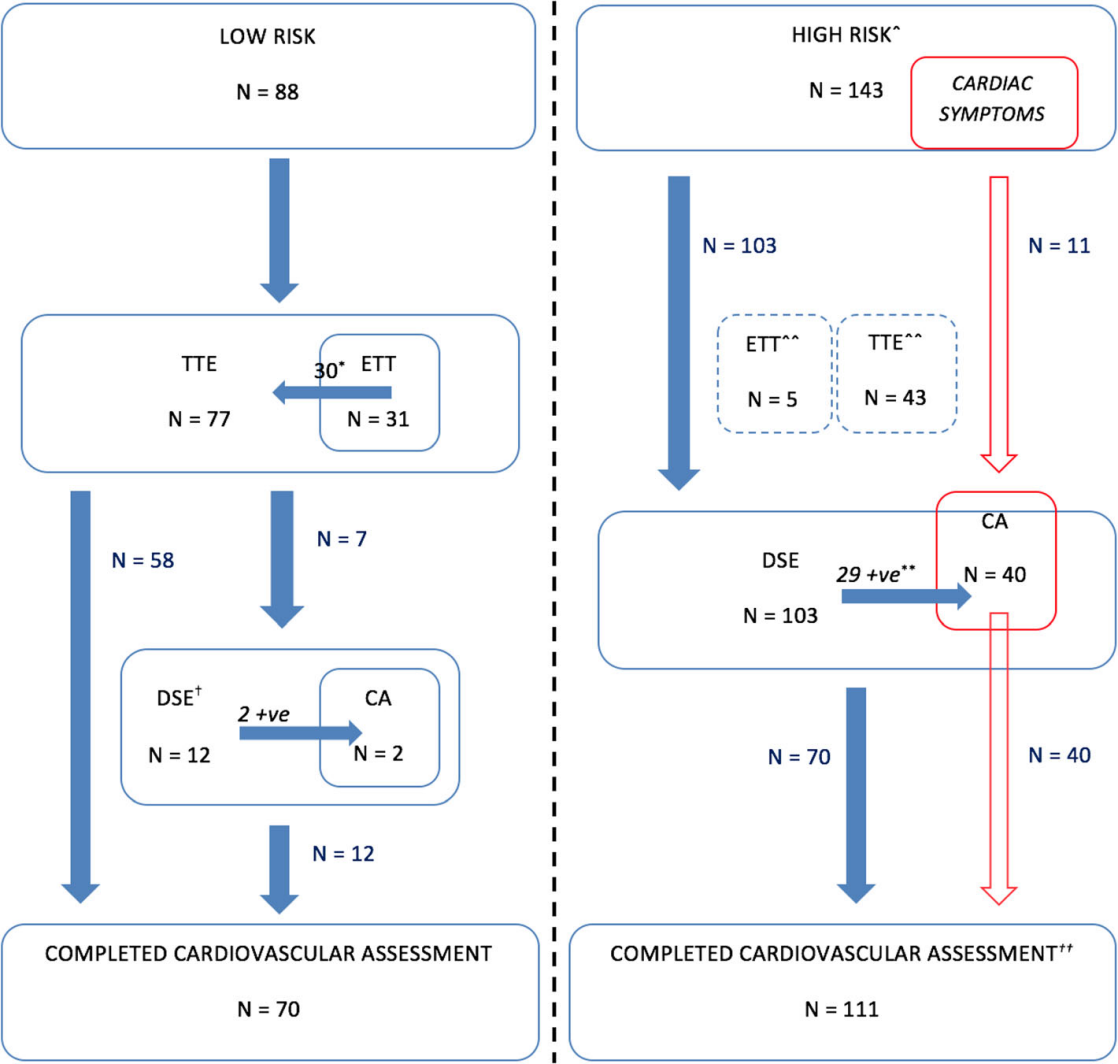
Risk stratification and subsequent investigations for potential kidney transplant recipients belonging to low-risk and high-risk groups. N = number of patients**.** ^*^1 patient awaiting TTE; ^†^5 patients had DSE without ETT or TTE (unable to perform ETT); ˆ29 patients deemed unsuitable for transplantation and discontinued further cardiac workup; ˆˆETT and TTE performed for other reasons outside of protocol; ^**^2 patients with positive DSE yet to complete CA; ^*††*^1 patient had a treadmill exercise echocardiography as DSE could not be performed; TTE, Transthoracic Echocardiography; ETT, Exercise Treadmill Test; DSE, Dobutamine Stress Echocardiography; CA, Coronary Angiography

Two hundred thirty-one patients were evaluated and mean length of follow up was 579 ± 289 days. One hundred forty-three patients were deemed ‘high-risk’. As shown in Table 1, these patients were older, had a higher BMI and were more likely to have cardiovascular risk factors. In total, 115 patients underwent a DSE, 42 CA, 31 ETT and 77 TTE. Figure 2 shows the number of patients belonging to the risk stratification groups adopted for this study and their subsequent investigations.

**Table 1:** Baseline characteristics of prospective renal transplant recipients undergoing cardiovascular risk assessment

	High Risk	Low Risk	
Number	143 (61.9%)	88 (38.1%)	
Age	59.9 ± 9.9	42.9 ± 9.8	*p* = 0.000
Male	81 (56.6%)	53 (60.2%)	*p* = 0.592
Body Mass Index (Kg/m^2^)	28.6 ± 5.4	26.4 ± 4.9	*p* = 0.003
Cholesterol (mmol/L)	4.11 ± 1.20	4.47 ± 1.22	*p* = 0.037
High Density Lipoprotein (mmol/L)	1.21 ± 0.47	1.28 ± 0.44	*p* = 0.317
Triglycerides (mmol/L)	1.68 ± 0.84	1.75 ± 1.16	*p* = 0.668
Parathyroid Hormone Level (pmol/L)	42.1 ± 48.9	40.5 ± 38.9	*p* = 0.786
C-Reactive Protein (mg/L)	9.4 ± 14.8	9.0 ± 14.7	*p* = 0.834
Haemoglobin (g/L)	107.8 ± 18.5	109.9 ± 15.9	*p* = 0.391
Ferritin (μg/L)	302.9 ± 293.9	250.5 ± 323.8	*p* = 0.211
Modality			*p* = 0.121
Haemodialysis	54 (37.8%)	28 (31.8%)	
Peritoneal Dialysis	4 (2.8%)	3 (3.4%)	
Kidney Transplant	8 (5.6%)	13 (14.8%)	
No Renal Replacement Therapy	77 (53.8%)	44 (50.0%)	
Diabetes	80 (55.9%)	0 (0%)	*p* = 0.000
Hypertension	135 (94.4%)	76 (86.4%)	*p* = 0.035
Ischaemic Heart Disease	29 (20.3%)	2 (2.3%)	p = 0.000
Ever smoked	54 (37.8%)	31 (35.2%)	*p* = 0.481
Peripheral Vascular Disease	4 (2.8%)	0 (0%)	*p* = 0.113
Cerebrovascular Accident/TIA	15 (10.5%)	1 (1.1%)	*p* = 0.007
Congestive Cardiac Failure	5 (3.5%)	0 (0%)	*p* = 0.076
ACEi/ARB	65 (45.5%)	34 (38.6%)	*p* = 0.309
Antiplatelet	54 (37.8%)	5 (5.7%)	*p* = 0.000
Beta-Blocker	43 (30.1%)	27 (30.7%)	*p* = 0.922
Statin	76 (53.1%)	18 (20.5%)	*p* = 0.000
Average length of follow up (days)	605.7 ± 294.8	568.5 ± 285.0	*p* = 0.346

Results are expressed as mean ± standard deviation or number and percentage (%) where indicated. *ACEi* Angiotensin Converting Enzyme Inhibitor, *ARB* Angiotensin II Receptor Blocker, *Statin* HMG CoA Reductase Inhibitor

At the end of follow-up, pre-transplant cardiac assessment was completed in 181 patients (70 in ‘low-risk’ and 111 in ‘high-risk’ groups); 50 patients were active on the transplant wait-list (24 ‘low-risk’ and 26 ‘high-risk’) and 35 had received a transplant.

### Events during follow up

There were a total of 24 events in 21 (9.1%) patients - none in the perioperative period (Table 2). The mean length of time to first event was 354 ± 197 days. The overall AER was 5.7% per year. Table 3 shows the characteristics of the patients who had and did not have events. Patients who had events were older, more likely to have diabetes, ischaemic heart disease and were more likely to be on typical cardiac medications. Unsurprisingly, all but one had been stratified as ‘high-risk’ (Table 3).

**Table 2:** Breakdown of events during follow-up

Event	Number (%)
Acute Coronary Syndrome	8 (30.7%)
Congestive Cardiac Failure	2 (7.7%)
Stroke/Transient Ischaemic Attack	3 (11.5%)
Amputation	2 (7.7%)
Death	9 (34.6%)
-Sudden Cardiac Death	2 (7.7%)

Results are expressed as total number and percentage. Events were defined as the occurrence after referral for transplant evaluation of a non-fatal cardiovascular event (myocardial infarction, unstable angina, congestive heart failure, stroke, transient ischaemic attack and amputation) and sudden cardiac death or death from any cause

**Table 3:** Baseline characteristics of patients having events compared to those without

	Event (*n* = 21)	No Event (*n* = 210)	
Age	59.9 ± 11.1	52.8 ± 12.9	*p* = 0.015
Male	12 (57.1%)	122 (58.1%)	*p* = 0.933
High Cardiovascular Risk^a^	20 (95.2%)	123 (58.6%)	*p* = 0.001
Body Mass Index (Kg/m^2^)	27.7 ± 4.7	27.8 ± 5.4	*p* = 0.948
Cholesterol (mmol/L)	3.67 ± 1.33	4.31 ± 1.19	*p* = 0.024
High Density Lipoprotein (mmol/L)	1.03 ± 0.34	1.25 ± 0.47	*p* = 0.054
Triglycerides (mmol/L)	1.49 ± 1.04	1.73 ± 0.96	*p* = 0.319
Parathyroid Hormone Level (pmol/L)	54.1 ± 56.3	40.2 ± 43.9	*p* = 0.182
C-Reactive Protein (mg/L)	14.6 ± 18.8	8.7 ± 14.2	*p* = 0.174
Haemoglobin (g/L)	106.8 ± 17.7	108.9 ± 17.6	*p* = 0.612
Ferritin (μg/L)	340.5 ± 474.7	276.8 ± 284.4	*p* = 0.365
Modality			*p* = 0.300
Haemodialysis	8 (38.1%)	74 (35.2%)	
Peritoneal Dialysis	0 (0%)	7 (3.3%)	
Kidney Transplant	4 (19.0%)	17 (8.1%)	
No Renal Replacement Therapy	9 (42.9%)	112 (53.3%)	
Diabetes	12 (57.1%)	68 (32.4%)	*p* = 0.023
Hypertension	20 (95.2%)	191 (91.0%)	*p* = 0.505
Ischaemic Heart Disease	10 (47.6%)	21 (10.0%)	*p* = 0.000
Ever smoked	9 (42.9%)	76 (36.2%)	*p* = 0.239
Peripheral Vascular Disease	0 (0%)	4 (1.9%)	*p* = 0.523
Cerebrovascular Accident/TIA	4 (19.0%)	12 (5.7%)	*p* = 0.022
Congestive Cardiac Failure	1 (4.8%)	4 (1.9%)	*p* = 0.391
ACEi/ARB	15 (71.4%)	84 (40.0%)	*p* = 0.006
Antiplatelet	15 (71.4%)	44 (21.0%)	*p* = 0.000
Beta-Blocker	11 (52.4%)	59 (28.1%)	*p* = 0.021
Statin	15 (71.4%)	79 (37.6%)	*p* = 0.003

Results are expressed as mean ± standard deviation or number and percentage (%) where indicated. Events were defined as the occurrence after referral for transplant evaluation of a non-fatal cardiovascular event (myocardial infarction, unstable angina, congestive heart failure, stroke, transient ischaemic attack and amputation) and sudden cardiac death or death from any cause

^**a**^High Cardiovascular Risk according to our risk stratification protocol. *ACEi* Angiotensin Converting Enzyme Inhibitor, *ARB* Angiotensin II Receptor Blocker, *Statin* HMG CoA Reductase Inhibitor

A total of 9 people died during the follow-up period. Eight had been stratified as ‘high risk’. Causes of death included myocardial infarction (1), intracerebral haemorrhage (1), sepsis (2), metatstatic cancer (1), cardiac arrest of unknown cause (2) and unknown (2). One patient belonged to the ‘low-risk’ cohort and died following an intracerebral haemorrhage.

There were no events in the 35 patients who received a kidney transplant during follow up. Of these, 18 were high risk, 21 had DSE, 1 had CA, none had significant CAD and none underwent preoperative coronary intervention.

### Patients undergoing Dobutamine stress echocardiography

Although there were 143 patients in the high-risk group, only 103 of these patients had a DSE. Eleven patients went straight to coronary angiography thus bypassing the need for DSE. The remaining 29 did not have a DSE as they were deemed unsuitable for transplantation at this stage (outstanding medical issues; stable eGFR) or for the following reasons: patient undecided, missed appointments, transferred to other unit or died.

Twelve low-risk patients had a DSE following an abnormal TTE (e.g. regional wall motion abnormalities and/or left ventricular dysfunction), positive or inconclusive ETT or were unable to do an ETT (e.g. poor mobility). Three of the 12 low-risk patients had a positive DSE of whom 2 had CA (1 awaiting CA) which did not reveal CAD and none of these 12 had events.

In total, 115 patients had a DSE of whom 34 (30%) were positive (i.e. ≥ 2 ischaemic segments). Table 4 shows the characteristics of patients who had a DSE. Patients with a positive DSE were more likely to have diabetes and ischaemic heart disease as well as already be on antiplatelet agents and HMG-CoA reductase inhibitors.

**Table 4:** Baseline characteristics of patients having Dobutamine Stress Echocardiography

	DSE positive	DSE negative	
Number	34	81	
Age	59.4 ± 8.2	58.8 ± 10.6	*p* = 0.736
Male	20 (58.8%)	46 (56.8%)	*p* = 0.841
Body Mass Index (Kg/m^2^)	28.2 ± 6.0	28.1 ± 4.1	*p* = 0.902
Cholesterol (mmol/L)	4.10 ± 1.16	4.28 ± 1.24	*p* = 0.491
High Density Lipoprotein (mmol/L)	1.21 ± 0.50	1.24 ± 0.43	*p* = 0.804
Triglycerides (mmol/L)	1.75 ± 1.00	1.51 ± 0.69	*p* = 0.179
Parathyroid Hormone Level (pmol/L)	31.6 ± 36.3	43.3 ± 52.3	*p* = 0.180
C-Reactive Protein (mg/L)	9.9 ± 12.0	9.4 ± 16.1	*p* = 0.867
Haemoglobin (g/L)	107.8 ± 15.3	107.8 ± 17.0	*p* = 0.998
Ferritin (μg/L)	292.6 ± 225.2	321.0 ± 329.4	*p* = 0.652
Modality			*p* = 0.313
Haemodialysis	14 (41.2%)	26 (32.1%)	
Peritoneal Dialysis	2 (5.9%)	1 (1.2%)	
Kidney Transplant	2 (5.9%)	9 (11.1%)	
No Renal Replacement Therapy	16 (47.1%)	45 (55.6%)	
Diabetes	24 (70.6%)	33 (40.7%)	*p* = 0.003
Hypertension	33 (97.1%)	77 (95.1%)	*p* = 0.632
Ischaemic Heart Disease	9 (26.5%)	7 (8.6%)	*p* = 0.012
Ever smoked	12 (35.3%)	35 (43.2%)	*p* = 0.594
Peripheral Vascular Disease	2 (5.9%)	2 (2.5%)	*p* = 0.362
Cerebrovascular Accident/TIA	4 (11.8%)	7 (8.6%)	*p* = 0.603
Congestive Cardiac Failure	1 (2.9%)	0 (0%)	*p* = 0.121
Medication			
ACEi/ARB	17 (50.0%)	39 (48.1%)	*p* = 0.856
Antiplatelet	19 (55.9%)	25 (30.9%)	*p* = 0.012
Beta-Blocker	15 (44.1%)	26 (32.1%)	*p* = 0.219
Statin	23 (67.6%)	36 (44.4%)	*p* = 0.023
High Cardiovascular Risk	31 (91.2%)	72 (88.9%)	*p* = 0.714

Results are expressed as mean ± standard deviation or number and percentage (%) where indicated. *DSE* Dobutamine Stress Echocardiography, *ACEi* Angiotensin Converting Enzyme Inhibitor, *ARB* Angiotensin II Receptor Blocker, *Statin* HMG CoA Reductase Inhibitor

Thirty patients had both DSE followed by CA (26 DSE positive). Out of the 26 patients with a positive DSE, 16 (62%) patients were found to have significant CAD (≥ 50% stenosis) on CA. Four patients with negative DSE had CA for the following reasons: severely impaired left ventricular function on DSE (1), acute coronary syndrome after DSE (2) and multiple cardiovascular risk factors (1). All 4 patients were found to have significant CAD with 3 requiring PCI and 1 referred for CABG. Three of these 4 patients had events (2 ACS and 1 death from sepsis). One patient who had an ACS was awaiting CABG.

**Fig. 3:**
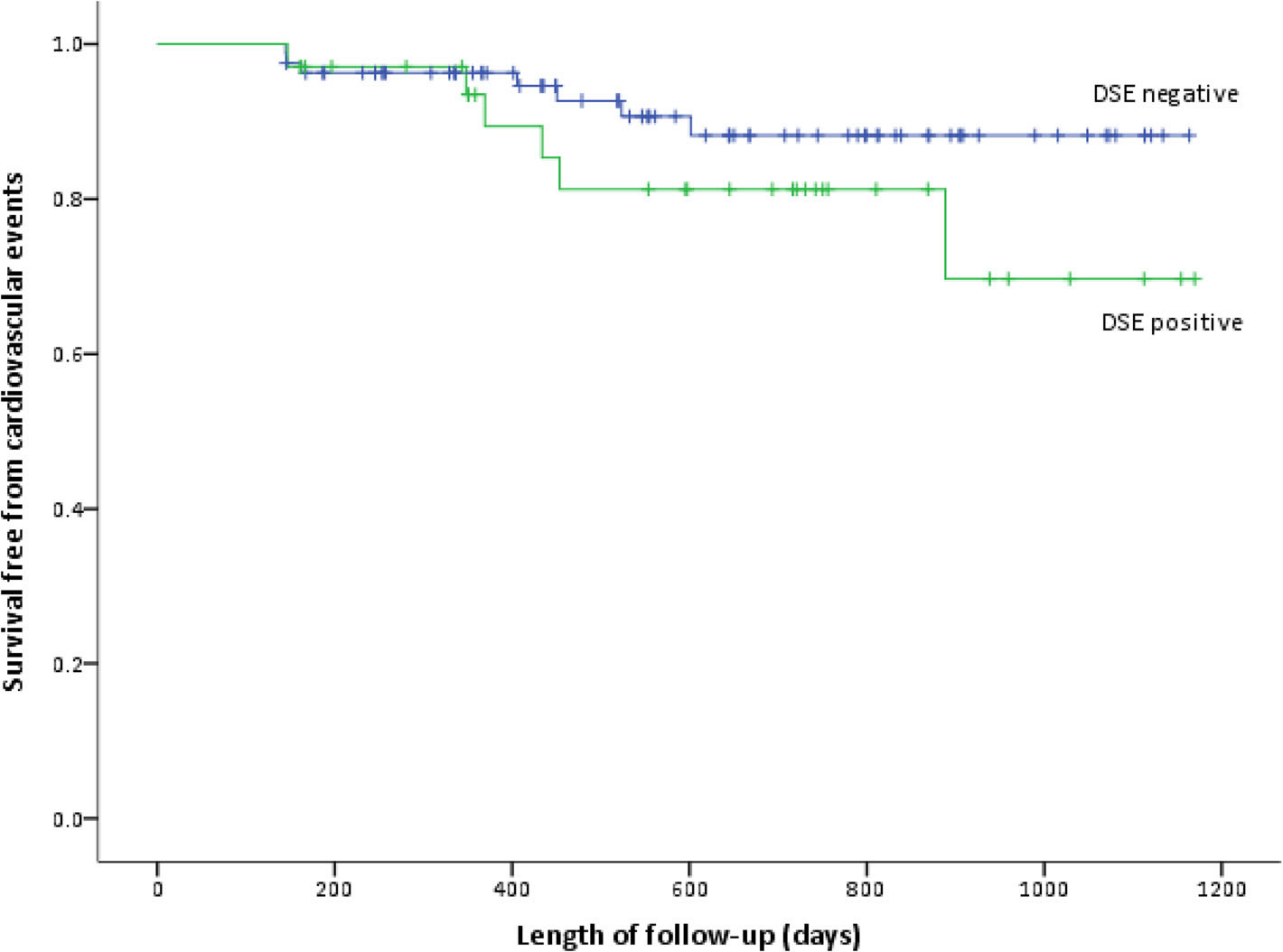
Event rates in patients with and without a positive dobutamine stress echocardiography test. DSE positive patients had more events compared to DSE negative patients (log rank *p* = 0.164). Events were defined as the occurrence after referral for transplant evaluation of a non-fatal cardiovascular event (myocardial infarction, unstable angina, congestive heart failure, stroke, transient ischaemic attack and amputation) and sudden cardiac death or death from any cause. DSE, Dobutamine Stress Echocardiography; CV, Cardiovascular

There were numerically more events but no statistical difference in patients with a positive DSE i.e. Six of 34 patients (AER 11.1%) compared to 7 of 81 patients (AER 5.4%) with a negative DSE (Pearsons Chi-square *p* = 0.164). Patients who had a positive DSE had 94% and 85% event-free survival at 1 and 2 years respectively compared to 96% and 91% in those patients with a negative DSE (log rank *p* = 0.193, Fig. 3). Similarly, using a Cox proportional hazard analysis, the risk for events was not significantly different between DSE positive patients compared to DSE negative patients (HR = 0.573, 95% CI 0.093 – 3.527, *p* = 0.549).

### Patients undergoing coronary angiography

Forty-two patients had CA of whom 30 (71.4%) were found to have significant CAD. Eighteen of 42 (42.9%) patients went on to have PCI or were referred for CABG and 13 patients had events (AER 19.5%). Events were predominantly ACS (8), but also included stroke or TIA (1), amputation (1) and all cause deaths (4). One death was due to ischaemic heart disease, 2 due to sepsis and 1 due to an unidentifiable cause. Seven patients were referred for CABG. Three of these 7 patients had had CABG during follow up. There were 2 events in 2 patients awaiting CABG (1 death of unknown cause and 1 ACS). Overall there were 6 ACS, 1 stroke and 1 death in the patients requiring coronary intervention.

**Fig. 4:**
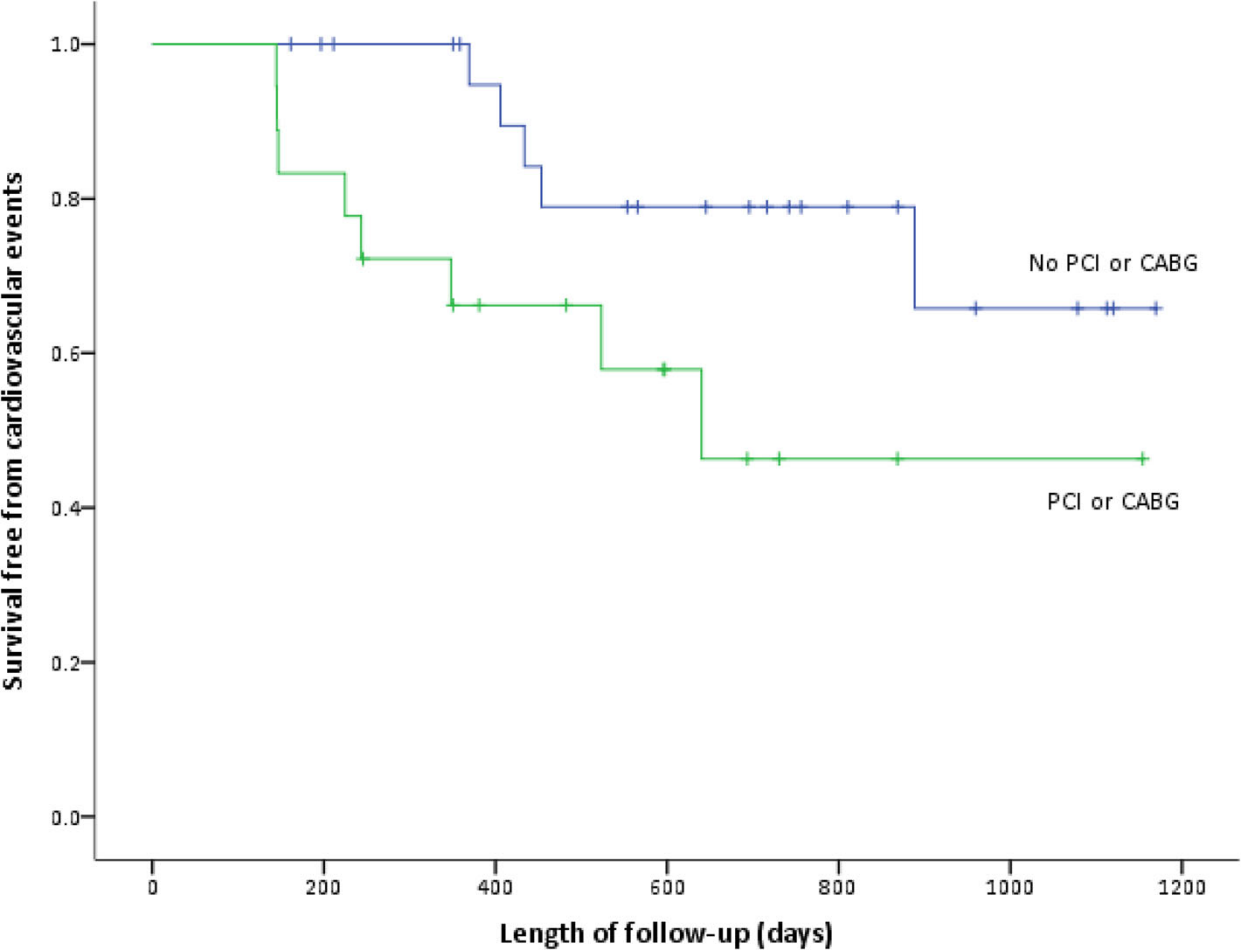
Event rates during follow-up in patients having had coronary angiography - comparing those requiring or not requiring PCI or CABG. Patients requiring PCI or CABG were more likely to have events (log rank *p* = 0.044). Events were defined as the occurrence after referral for transplant evaluation of a non-fatal cardiovascular event (myocardial infarction, unstable angina, congestive heart failure, stroke, transient ischaemic attack and amputation) and sudden cardiac death or death from any cause. PCI, Percutaneous Coronary Intervention; CABG, Coronary Artery Bypass Graft

Event rates were numerically higher but statistically not significant in patients requiring coronary artery intervention i.e. PCI or CABG (8 out of 18, AER 28.0%) compared to those who did not require intervention (5 out of 24, AER 13.1%; Pearson Chi-square *p* = 0.101). Figure 4 shows the Kaplan-Meier event-free survival rates between the 2 groups. Patients not requiring intervention had significantly better event-free survival at 1 and 2 years when compared to those patients with significant CAD requiring coronary intervention (100% and 83% vs 67% and 56%, log rank *p* = 0.044). However, using a cox proportional hazard analysis, the risk for events was no longer significantly different (HR = 3.17, 95% CI 0.512 – 19.591, *p* = 0.215).

Eleven patients had CA without a prior DSE (10 for cardiac symptoms and 1 for impaired left ventricular function in association with significant cardiac risk factors) and 6 of these 11 were found to have significant CAD requiring intervention (2 PCI and 4 referred for CABG). Two patients had acute coronary syndrome events and 1 died of an unknown cause.

In the 42 patients who had CA, only 1 patient with mild (<50% stenosis) single-vessel CAD received a transplant (no events at end of the follow-up period), 3 patients were active on the transplant waitlist, 6 were suspended (1 awaiting CABG, 2 on dual-anti-platelets, 1 awaiting CA after repeat DSE was positive and 2 undergoing further medical investigations), 11 were removed from waitlist (10 medically unfit, 1 declined CABG),17 are still undergoing assessment, 1 was transferred to another centre and 4 died.

### Low risk patients

Eighty-eight patients were stratified as low-risk. Thirty-one had ETT and 77 had TTE. Twelve patients had DSE for abnormal/suboptimal ETT or abnormal TTE (7) or were unable to do an ETT (5). Three DSEs were positive and 2 subsequently had CA which revealed no CAD (1 CA pending). None of the 5 DSEs done without a prior ETT or TTE were positive. There was only 1 event in the low-risk group which was a death caused by an intracerebral haemorrhage (AER 0.7%). Ten patients did not have an ETT or TTE (5 had DSE because of inability to perform an ETT, 1 waiting for DSE, 3 stable renal function and therefore further assessment currently postponed and 1 missed several appointments).

## Discussion

The major findings in this “real life” study of protocol driven CV work-up for all kidney transplant recipients were as follows. Firstly, risk stratification identified 143 (62%) potential renal transplant recipients as high-risk who underwent DSE and if necessary CA yet only 18 (12.6%) underwent coronary artery intervention or CABG. Secondly event rates in the low-risk group were minimal (AER 0.7%) indicating that clinical risk stratification was an effective tool to avoid unnecessary testing in these patients. Thirdly, 74% of cases with a positive DSE had CAD on CA suggesting a positive DSE was at least a fair predictor of CAD on CA.

The event rate amongst the low-risk patients in this study is very low (0.7% per year). Indeed, there was only one event in this group. The process of risk stratifying potential renal transplant recipients at the onset of the evaluation process correctly identifies those patients least likely to have cardiovascular events or death from any cause around the transplantation period and during follow-up. Unsurprisingly these patients are younger and less likely to have important cardiovascular risk factors such as previous ischaemic heart disease, diabetes and tend to have a lower BMI. However, we also recognise duration of follow-up for this group was 569 ± 285 days which when compared to previous studies is much shorter. Kasiske et al. [16] report an incidence of 0.5% in the first year for low-risk patients not screened for CV disease (13 patients had a coronary event out of 224 patients during a mean follow-up of 88 months) and Lewis et al. [17] reported 1 of 94 patients belonging to their low-risk group having a cardiac death during a mean follow-up of 28 months. In another study of 600 patients undergoing renal transplantation, Patel et al [18]. reported 19/426 patients in their low-risk group having CV events over a mean follow-up of 42 months. The mean length of follow-up for this study was 579 ± 289 days and a longer follow-up period may yield more events, but the available data suggest the event rates will remain low in a low CV risk group. Transplantation may further improve the CV risk profile for these patients thus they should be considered for activation on the kidney transplant waitlist without further delay in keeping with ACC/AHA guidelines for perioperative CV evaluation in non-cardiac surgery and European Renal Best Practice Guidance [21–25].

Contrary to the above, event rates occur more frequently in patients with high CV risk and in our study all but one event were in the high-risk group (23 events in 20 patients out of 143 patients; AER 8.9%). Several studies have evaluated the appropriateness of DSE in evaluation of CAD in the general population [26–28] and in patients with ESRD [29, 30]. In a relatively small study of 50 renal transplant candidates, Herzog et al. [29] reported high sensitivity, specificity, positive predictive value and negative predictive value of DSE in predicting CAD; 52%, 74%, 70% and 57% respectively for coronary artery stenosis of 50%; and 75%, 71%, 45% and 90% respectively for stenosis greater than 70%. Similarly, Sharma et al. [27] reported sensitivity, specificity, positive predictive value and negative predictive value of 88%, 94%, 86% and 95% respectively in detecting coronary artery disease (stenosis >70%) in patients with ESRD. Admittedly the two studies are small and evidence may not be generalisable. In this study, 62% of all patients with a positive DSE were found to have significant CAD on CA. Four patients with a negative DSE had a CA and all were found to have significant CAD (3 requiring coronary intervention). These patients belonged to the high-risk group and the indications for CA were strongly suggestive of unstable CAD (2 ACS, 1 severely impaired left ventricular function and multiple CV risk factors with previous ACS and PCI). Thus, the occurrence of symptoms even after negative DSE merit further invasive investigations.

Our study relies on DSE as a non-invasive test, which is supervised by a single operator who is a cardiologist with an interest in the pre-transplant population. However other non-invasive tests have been utilised such as MPS, PET and SPECT. DSE, MPS and SPECT have similar sensitivities in detecting coronary artery disease [31]. PET scanning has the ability to show coronary flow reserve in addition to ischaemia for better prediction of adverse outcomes. However, most centres rely on DSE or MPS [31].

In the present study, there was a non-significant trend toward worse event-free survival in patients with a positive DSE compared to patients with a negative DSE (Fig. 3). This is consistent with Herzog et al. [29] who reported 6 events (20%) in 30 negative DSE patients and 11 (55%) events in 20 patients with positive DSE with an average follow-up of 22 ± 10 months.

In the present study, CA was offered to patients with a significant ischaemic burden on DSE with the goal to identify angiographic significant CAD lesions and offer definitive treatment as appropriate. The majority underwent PCI and a few (7) were referred and even fewer (3) had CABG during follow-up. The decision for CA intervention was taken by the interventional cardiologist at the time of angiography based on visual determination of CAD stenosis and was therefore a subjective decision. Pressure-wire studies were occasionally used in borderline cases. The goal of pre-emptive revascularisation was to reduce risk of CV events perioperatively and allow activation on the waitlist. However, there was significantly worse event-free survival in patients with significant CAD requiring intervention compared to those not requiring intervention. This observation is supported by Herzog et al. [29] who reported significantly worse cardiac event-free survival in patients with at least one stenosis ≥ 50% and Patel et al. [19] who reported 99 patients undergoing CA with 17 undergoing PCI or CABG and no significant difference in mortality between those patients undergoing PCI or CABG compared to those who underwent CA without intervention or no CA. Thus the results of screening were important predictors of survival, but did not lead to event-free survival benefit with intervention and perhaps did little more than exclude some patients from transplantation. This is in keeping with trials in the non-ESRD population which have shown that prophylactic coronary artery revascularization in asymptomatic patients does not reduce all-cause mortality or improve outcomes in high-risk patients undergoing major non-cardiac surgery [6, 32]. Overall there were 6 ACS, 1 stroke and 1 death in the patients requiring coronary intervention. This is more likely to be a reflection of high CV disease burden and coexisting comorbidity amongst these patients and suggests these most-at-risk patients were correctly identified in the pre-transplant assessment.

Thus, there is little evidence in support of more invasive CV assessment with CA and coronary intervention in patients evaluated for kidney transplantation. The case for CA for all, however, has been put forward by Kumar et al. [20] where cardiac event-free survival amongst those who underwent intervention (*n* = 168) was particularly high, 98% and 88% at 1 and 3 years respectively as opposed to 75% and 35% in patients who declined intervention (*n* = 16, with similar baseline characteristics) with 10 of 16 deaths attributed to a cardiac cause. Only 1 of 20 (5%) and 1 of 30 (3.3%) of their patients died of CV causes whilst on the waitlist or after transplantation respectively. Although there were no reported complications of CA including decline in renal function requiring premature renal replacement therapy in this study, this remains a concern when considering CA. Whether coronary intervention in CKD patients with moderate ischaemia on non-invasive stress testing is useful will be addressed in the ongoing ISCHAEMIA- Chronic Kidney Disease trial (NCT01985360) due to report in 2019.

The limitations of this study includes a short follow-up period resulting in fewer cardiovascular events overall. However, the main conclusions of this study are unlikely to change significantly with a longer follow-up as suggested by the available literature. Some CV risk factors such as dialysis duration and length of diabetes were not included in the data analysis as this data was not available. Although a few patients did not follow the protocol strictly, this did not alter their pathway through the CV assessment and is unlikely to have a significant impact on the results of this study. An observational study such as this to investigate impact of coronary intervention in asymptomatic patients with CAD has significant limitations due to lack of randomisation and a parallel non-intervention group.

Given that, coronary artery intervention in asymptomatic individuals with CAD is not recommended in the general population [6–8], the current practice for screening these asymptomatic patients prior to transplantation is questionable especially as there has been no conclusive evidence of benefit in the studies reported to date. However, with cardiovascular event rates and death from cardiovascular disease a leading cause of morbidity and mortality following transplantation [10–15], this practice has been widely adopted in the transplant community in the belief that recognition and intervention will result in lower cardiovascular events and death.

The study demonstrates that the standardised protocol is successful in identifying the patient with high risk, and non-invasive testing identifying the patients at highest risk, however the role of intervention is not clear as these patients remained the most at risk. It is for these highest risk patients that a randomised control trial is required to identify the few patients who may benefit from intervention. Renal transplantation confers better long-term survival and quality of life compared to patients awaiting transplantation [9] and therefore should be available to all suitable prospective recipients. Until better evidence for cardiovascular disease risk management emerges, the decision for coronary revascularisation, optimisation of medical management and transplantation should be made on a case by case basis and involve transplant and nephrology, cardiology and cardiothoracic teams.

## Conclusion

The study suggests that using a standardised protocol to identify high-risk patients with DSE for screening is effective at identifying those patients with coronary artery disease in a cohort referred for kidney transplantation. The coronary angiogram triggered by positive DSE or clinical symptoms, correctly identifies the patients likely to suffer from death and CV disease during follow-up but coronary intervention does not seem to alter prognosis. Given CV disease is a leading cause of morbidity and mortality in potential transplant recipients on the waitlist and after transplantation, the role of coronary angiography and coronary intervention which are costly, not without risk and generously employed in this context need to be evaluated in prospective randomised trials.

## Abbreviations

ACS: Acute Coronary Syndrome
AER: Annual Event Rate
BMI: Body Mass Index
CA: Coronary Angiography
CABG: Coronary Artery Bypass Graft
CAD: Coronary Artery Disease
CV: Cardiovascular
DSE: Dobutamine Stress Echocardiography
eGFR: Estimated Glomerular Filtration Rate
ETT: Exercise Treadmill Test
HR: Hazard Ratio
MPS: Myocardial Perfusion Scan
PET: Positron Emission Tomography
RRT: Renal Replacement Therapy
SPECT: Single-Photon Emission Computed Tomography
TIA: Transit Ischaemic Attack
TTE: Transthoracic Echocardiography

## References

[1] Nakano T, Ninomiya T, Sumiyoshi S (2010). Association of kidney function with coronary atherosclerosis and calcification in autopsy samples from Japanese elders: the Hisayama study. Am J Kidney Dis.

[2] Chonchol M, Whittle J, Desbien A (2008). Chronic kidney disease is associated with angiographic coronary artery disease. Am J Nephrol.

[3] Fishbane S (2005). Cardiovascular risk evaluation before kidney transplantation. J Am Soc Nephrol.

[4] Ohtake T, Kobayashi S, Moriya H (2005). High prevalence of occult coronary artery stenosis in patients with chronic kidney disease at the initiation of renal replacement therapy: an angiographic examination. J Am Soc Nephrol.

[5] Hayashi T, Obi Y, Kimura T (2008). Cardiac troponin T predicts occult coronary artery stenosis in patients with chronic kidney disease at the start of renal replacement therapy. Nephrol Dial Transplant.

[6] McFalls EO, Ward HB, Moritz TE (2004). Coronary-artery revascularization before elective major vascular surgery. N Engl J Med.

[7] Wijeysundera HC, Ko DT (2009). Does percutaneous coronary intervention reduce mortality in patients with stable chronic angina: are we talking about apples and oranges?. Circ Cardiovasc Qual Outcomes.

[8] Boden WE, O'Rourke RA, Teo KK (2007). Optimal medical therapy with or without PCI for stable coronary disease. N Engl J Med.

[9] Wolfe RA, Ashby VB, Milford EL (1999). Comparison of mortality in all patients on dialysis, patients on dialysis awaiting transplantation, and recipients of a first cadaveric transplant. N Engl J Med.

[10] Lentine KL, Brennan DC, Schnitzler MA (2005). Incidence and predictors of myocardial infarction after kidney transplantation. J Am Soc Nephrol.

[11] Kasiske BL, Maclean JR, Snyder JJ (2006). Acute myocardial infarction and kidney transplantation. J Am Soc Nephrol.

[12] Lentine KL, Schnitzler MA, Abbott KC (2005). De novo congestive heart failure after kidney transplantation: a common condition with poor prognostic implications. Am J Kidney Dis.

[13] Lentine KL, Schnitzler MA, Abbott KC (2006). Incidence, predictors, and associated outcomes of atrial fibrillation after kidney transplantation. Clin J Am Soc Nephrol.

[14] Ramanathan V, Goral S, Tanriover B (2005). Screening asymptomatic diabetic patients for coronary artery disease prior to renal transplantation. Transplantation.

[15] Morales JM, Marcen R, del Castillo D (2012). Risk factors for graft loss and mortality after renal transplantation according to recipient age: a prospective multicentre study. Nephrol Dial Transplant.

[16] Kasiske BL, Malik MA, Herzog CA (2005). Risk-stratified screening for ischemic heart disease in kidney transplant candidates. Transplantation.

[17] Lewis MS, Wilson RA, Walker KW (2002). Validation of an algorithm for predicting cardiac events in renal transplant candidates. Am J Cardiol.

[18] Patel AD, Abo-Auda WS, Davis JM (2003). Prognostic value of myocardial perfusion imaging in predicting outcome after renal transplantation. Am J Cardiol.

[19] Patel RK, Mark PB, Johnston N (2008). Prognostic value of cardiovascular screening in potential renal transplant recipients: a single-center prospective observational study. Am J Transplant.

[20] Kumar N, Baker CS, Chan K (2011). Cardiac survival after pre-emptive coronary angiography in transplant patients and those awaiting transplantation. Clin J Am Soc Nephrol.

[21] European Renal Best Practice Transplantation Guideline Development Group (2013). ERBP guideline on the management and evaluation of the kidney donor and recipient. Nephrol Dial Transplant.

[22] Dudley C, Harden P (2011). Renal association clinical practice guideline on the assessment of the potential kidney transplant recipient. Nephron Clin Pract.

[23] Kalble T, Lucan M, Nicita G (2005). EAU guidelines on renal transplantation. Eur Urol.

[24] Kasiske BL, Cangro CB, Hariharan S (2001). The evaluation of renal transplantation candidates: clinical practice guidelines. Am J Transplant.

[25] Fleisher LA, Beckman JA, Brown KA (2007). ACC/AHA 2007 guidelines on Perioperative cardiovascular evaluation and Care for Noncardiac Surgery: executive summary: a report of the American College of Cardiology/American Heart Association task force on practice guidelines (writing committee to revise the 2002 guidelines on Perioperative cardiovascular evaluation for noncardiac surgery) developed in collaboration with the American Society of Echocardiography, American Society of Nuclear Cardiology, Heart Rhythm Society, Society of Cardiovascular Anesthesiologists, Society for Cardiovascular Angiography and Interventions, Society for Vascular Medicine and Biology, and Society for Vascular Surgery. J Am Coll Cardiol.

[26] Senior R, Monaghan M, Becher H (2005). Stress echocardiography for the diagnosis and risk stratification of patients with suspected or known coronary artery disease: a critical appraisal. Supported by the British Society of Echocardiography. Heart.

[27] Senior R, Khattar R, Lahiri A (1998). Value of dobutamine stress echocardiography for the detection of multivessel coronary artery disease. Am J Cardiol.

[28] Marwick T, Willemart B, D'Hondt AM (1993). Selection of the optimal nonexercise stress for the evaluation of ischemic regional myocardial dysfunction and malperfusion. Comparison of dobutamine and adenosine using echocardiography and 99mTc-MIBI single photon emission computed tomography. Circulation.

[29] Herzog CA, Marwick TH, Pheley AM (1999). Dobutamine stress echocardiography for the detection of significant coronary artery disease in renal transplant candidates. Am J Kidney Dis.

[30] Sharma R, Pellerin D, Gaze DC (2005). Dobutamine stress echocardiography and the resting but not exercise electrocardiograph predict severe coronary artery disease in renal transplant candidates. Nephrol Dial Transplant.

[31] Bhatti NK, Karimi Galougahi K, Paz Y, et al. Diagnosis and Management of Cardiovascular Disease in advanced and end-stage renal disease. J Am Heart Assoc. 2016;5 doi:10.1161/JAHA.116.003648. 10.1161/JAHA.116.003648 PMC5015288 27491836

[32] Schouten O, van Kuijk JP, Flu WJ (2009). Long-term outcome of prophylactic coronary revascularization in cardiac high-risk patients undergoing major vascular surgery (from the randomized DECREASE-V pilot study). Am J Cardiol.

